# Analysis of an outpatient child hearing health program: from screening to referral for rehabilitation

**DOI:** 10.1590/2317-1782/20212020403

**Published:** 2022-02-02

**Authors:** Kátia de Cássia Botasso, Maria Cecília Marconi Pinheiro Lima, Carlos Roberto Silveira Correa

**Affiliations:** 1 Departamento de Saúde Coletiva, Faculdade de Ciências Médicas, Universidade Estadual de Campinas – UNICAMP - Campinas (SP), Brasil.; 2 Departamento de Desenvolvimento Humano e Reabilitação, Faculdade de Ciências Médicas, Universidade Estadual de Campinas – UNICAMP - Campinas (SP), Brasil.

**Keywords:** Neonatal Screening, Evaluation of Health Programs and Projects, Hearing Loss, Diagnosis, Rehabilitation

## Abstract

**Purpose:**

To analyze the stages of a hearing health program, from screening to referral for rehabilitation, based on the quality indicators for neonatal screening programs.

**Methods:**

This is a cohort, observational, retrospective study encompassing all newborns included in the Municipal Information System of Mogi Mirim, São Paulo, from 2010 to 2016. Besides the data present in the Information System on Live Newborns, the newborn’s age at the first test, test and diagnosis results, and referrals for rehabilitation were analyzed, based on the quality indicator criteria recommended by the Neonatal Hearing Screening Care Guidelines, with a statistical program.

**Results:**

A total of 7,800 newborns participated. The following results were obtained in the analysis of the program quality indicators: 1) Neonatal hearing screening stage: 97% coverage in the first test; 91% of newborns by 30 days old; 2) Diagnosis stage: 0.24% referred after failing the second test; 94.73% adherence; 13.66% confirmed diagnosis by 3 months old; 3) Rehabilitation stage: 100% began speech-language-hearing therapy immediately after the diagnosis; 20% received the hearing aid within 1 month from diagnosis.

**Conclusion:**

The program, conducted in an outpatient setting, met the recommendations of the guidelines presented by the Ministry of Health concerning the coverage and age at first examination, age at screening up to 1 month old, referral for diagnosis, and beginning the intervention. These results were obtained thanks to institutional support from the municipality.

## INTRODUCTION

Child hearing health programs are implemented to detect hearing loss on time, thus minimizing the difficulties resulting from sensory deprivation. To this end, both the Joint Committee on Infant Hearing (JCIH) and the Neonatal Hearing Screening (NHS) Care Guidelines of the Brazilian Ministry of Health proposed five stages for hearing health programs, namely: test, retest, diagnosis, rehabilitation, and monitoring and follow-up of auditory and language development^(1,2)^.

Federal law no. 12.303, of August 2, 2010, made free screening mandatory to detect hearing loss early with the evoked otoacoustic emissions at a hospital setting^(3)^.

In the municipality of Mogi Mirim, the debate about the importance of implementing a hearing health program took place in 2005. In that year, the health coordination presented a project of such a program to the municipal administrators. The proposal was approved in the following year, during the municipal health conference. Then, municipal law no. 12.522, of January 2, 2007, was passed, making it mandatory to perform the otoacoustic emissions screening in children of the municipality at outpatient centers^(4)^. The child hearing health program, in its turn, was implemented in 2009, after the municipal administration approved the acquisition of equipment and the training of the screening and rehabilitation teams. Therefore, social control and interface with the legislature were essential to implement the child hearing health program in the municipality.

The child hearing health program began instructing pregnant women in primary health care. It was implemented by a multiprofessional team, encompassing nurses, speech-language-hearing therapists, gynecologists, psychologists, nutritionists, and social workers. The speech-language-hearing therapists are responsible for instructing about the importance of breastfeeding, the consequences of deleterious habits, the importance of the stages of the hearing health program – test, retest, diagnosis, rehabilitation (speech-language-hearing therapy and hearing aid fitting), and auditory and language development monitoring and follow-up in our child population in primary health care.

The program performs the tests, retests, and rehabilitation (speech-language-hearing therapy) and monitors the development in the same municipality where the study was carried out. However, when diagnosis and hearing aid are necessary, the newborns are referred to the Regional Hospital in Divinolândia – Conderg, located in the municipality of Divinolândia, São Paulo, a reference center in hearing health.

The NHS Care Guidelines of the Ministry of Health proposed quality indicators to assess a child hearing health program, namely: 1) NHS stage: 95% coverage, aiming at 100%, up to 1 month old at the screening, and 2% to 4% referred for diagnosis; 2) Diagnosis stage: at least 90% attendance rate, and the diagnosis confirmed by 3 months old; 3) Rehabilitation stage: 95% of the newborns beginning speech-language-hearing therapy after reaching a diagnosis, and 95% of the newborns with permanent bilateral or unilateral hearing loss having received a hearing aid immediately after confirming the hearing loss^(2)^.

Many national studies analyze quality indicators of NHS programs. However, they normally use databases from one or more maternity hospitals, instead of the whole population encompassing all live newborns in a municipality. There is also research on some indicators proposed by the NHS Care Guidelines, such as age at first screening and diagnosis referral rate^(5-7)^.

This study is justified by its description of all the stages and quality indicators of a child hearing health program, providing a model for other teams that intend to start similar projects.

Given the above, the objective of this study was to analyze all the stages of a hearing health program, from the screening to the beginning of rehabilitation, following the quality indicator criteria for NHS programs.

## METHODS

The research project was submitted to the Research Ethics Committee of the *Universidade Estadual de Campinas* (State University of Campinas – UNICAMP) and approved on February 6, 2018, under evaluation report no. 2.487.739. However, the informed consent form was not required for this study because it was carried out with secondary data, previously collected, organized, and tabulated. Moreover, much of this information is public domain^(8)^.

This is a cohort, observational, retrospective study comprising all 7,800 participants registered in the Municipal Information System who took part in the child hearing health program in the municipality of Mogi Mirim between 2010 and 2016.

The first stage of the program involves the examination with transient evoked otoacoustic emissions (TEOAE), which became mandatory in 2007, performed at the outpatient Specialized Center of Mogi Mirim, which is where the municipal speech-language-hearing therapists carry out their activities. The hearing health program, though, was implemented in 2009, after the municipal administration approved the acquisition of equipment and the training of the screening and rehabilitation teams.

This research was based on secondary data, which are named this way for having been previously collected, organized, and tabulated, much of the information being public domain^(8)^. The data were obtained from a record book, implemented together with the hearing health program, making it the first database of the program.

This first database contains information on the participants who live in Mogi Mirim and were submitted to TEOAE at the Speech-Language-Hearing Outpatient Center, located at the Specialized Center of Mogi Mirim or in other institutions of the Information System on Live Newborns (SINASC, in Portuguese). It also contains the family sociodemographic variables; clinical history with risk indicators for hearing loss (RIHL); date and results of the examinations performed in the test, retest, and diagnosis; referrals; the date when they received the hearing aid; and beginning of the speech-language-hearing therapy.

Since 2015, this information was entered into a spreadsheet to make it easier to assess the quality indicators of the program. Hence, more data were made available regarding the pregnancy follow-up of the participants’ mothers and the program that monitored the auditory and language development.

The Maico Ero-Scan was the equipment used for the TEOAE examination, performed at 1.5 kHz to 4 kHz. The pass criteria were a signal-to-noise ratio higher than 4 dBSPL at 1 kHz and 6 dBSPL at the other frequencies, in at least three frequencies assessed, including 4 kHz, besides the presence of cochlear-palpebral reflex, obtained with agogo iron bells. The newborn was said to have passed the test and retest (i.e., not to have auditory changes) when the cochlear-palpebral reflex and TEOAE were present in both ears. On the other hand, when these auditory responses were not present, the newborn was said to have failed the screening.

All newborns who failed the first test were referred for the retest. If the failure persisted, acoustic immittance measures were taken (i.e., the tympanometry and the acoustic reflex testing). When the immittance measures were abnormal, according to the guideline manual for audiological assessments of the speech-language-hearing council system^(9)^, the participants were referred to an otorhinolaryngologist, and the TEOAE were reassessed.

However, when the immittance measures had a normal result (tympanometry type A curve and acoustic reflex present) but the newborn failed the TEOAE, they were referred to the examination of the brainstem auditory evoked potentials (BAEP), performed with the equipment Pentetek – Audtec – brainstem potential of the Audiscan system, which assesses with click stimulus the integrity of the auditory pathway at 2 to 4 kHz and the specific frequency of 1 kHz.

The newborn was said to have a RIHL when at least one of the following indicators proposed by the JCIH^(1)^ and the NHS Care Guidelines^(2)^ was present: the parents’ concern with the child’s auditory, speech, or language development; a family history of permanent deafness and consanguinity; an ICU stay for more than 5 days; mechanical ventilation use; exposure to ototoxic medications; severe perinatal anoxia; one-minute Apgar score of 0 to 4, or five-minute Apgar score of 0 to 6; birth weight lower than 1,500 grams; congenital infections (toxoplasmosis, rubella, cytomegalovirus, herpes, syphilis, HIV); craniofacial anomalies involving the ears and temporal bone; genetic syndromes; hyperbilirubinemia; neurodegenerative disorders; postnatal bacterial or viral infections (cytomegalovirus, herpes, measles, chickenpox, and meningitis); traumatic brain injury; chemotherapy^(1,2)^. Mother’s alcohol or psychotropic substance use during pregnancy, peri-intraventricular hemorrhage, and neonatal seizures were also included^(10)^. It is important to highlight that the RIHL used in this study are from the 2007 JCIH positioning statement^(1)^, as it was only reedited in 2019^(11)^ when the research was already concluded.

When the examination results were abnormal, the newborn was referred to the Regional Hospital of Divinolândia - Conderg to confirm the diagnosis, perform complementary examinations, and start using the hearing aid. Then, the family received, in the same municipality, the first instructions regarding rehabilitation. Lastly, all participants were referred to the child development monitoring and observation program in primary health care, with the reference speech-language-hearing therapist. This procedure has been used in the municipality for many years, in which all children have their global development followed up, including their auditory development, due to progressive or late-onset loss.

The following variables were approached to analyze the indicators in the first stage of the program: screening coverage, age in days at the first test, examination results in the test and retest, mean age at diagnosis. In the diagnosis stage, the following variables were analyzed: referral for diagnosis, attendance to the high-complexity service, infant’s age at confirmed diagnosis, confirmed diagnosis, types of loss, and prevalence of permanent and temporary losses. In the rehabilitation stage, the variables were as follows: referrals for hearing aid, age when they received the hearing aids and age at the beginning of the speech-language-hearing therapy.

The parameters suggested by the NHS Care Guidelines were used to analyze the quality indicators of the program^(2)^:

1. NHS stage: 95% coverage of live newborns who lived in the municipality; being up to 30 days old at the test.
2. Diagnosis stage: 2% to 4% referrals for diagnosis (at the high-complexity service); 90% attendance at the high-complexity service; diagnosis confirmed by 3 months old.
3. Rehabilitation stage: 95% of the candidates having received the hearing aid within 1 month from diagnosis; 95% of infants with a confirmed diagnosis of permanent bilateral or unilateral hearing loss having begun speech-language-hearing therapy immediately after confirming the diagnosis.

The frequency distribution of the categorical variables and the percentage values were descriptively analyzed. For the coverage, the mean and standard deviation were also used; and for age at the first TEOAE screening, quartiles 1, 2, and 3 were used. All data were analyzed with the SAS statistical program.

Lastly, the prevalence measure of permanent and temporary hearing loss was used, considering 1) the number of cases with permanent hearing loss during the time of the study in relation to the total number of participants during the time of the study; 2) the number of cases with temporary hearing loss during the time of the study in relation to the total number of participants during the time of the study.

## RESULTS

The information of the 7,800 participants of the child hearing health program from 2010 to 2016 was analyzed.

The data on the first TEOAE screening coverage of newborns who lived in the municipality are shown in Table 1. In the first year (2010), there was a 93% coverage, whereas the whole time of the study had a mean of 97.00% (SD: 0.02).

**Table 1:** Description of the quality indicator regarding the coverage of the first otoacoustic emission test in live newborns living in the municipality

Year of Birth	Resident Live Newborns	Frequency	Percentage
	N= 7,962	N= 7,800	%
2010	1,144	1,072	93.00
2011	1,089	1,075	98.00
2012	1,153	1,142	99.00
2013	1,168	1,123	96.00
2014	1,154	1,120	97.00
2015	1,157	1,151	99.00
2016	1,097	1,043	95.00
Mean: 97.00; Standard Deviation: 0.02			

Source: The researcher's own database.

Insert Table 1 here

Regarding the first stage of the test, 7,294 (93.51%) of the 7,800 participants passed, while 506 (6.49%) failed and were referred for the retest.

Of the participants who were referred for the retest, 460 (90.90%) attended it, of whom 348 (75.65%) passed and 112 (24.35%) continued with a failed result.

For BAEP screening, there were 1,049 referrals (13.48%) – 937 (89.32%) due to RIHL at birth, 56 (5.34%) for failing the TEOAE retest and having RIHL at birth, and 56 (5.34%) for failing the TEOAE retest though not having RIHL at birth. Of these, 738 (70.48%) attended the screening – 505 of them (48.23%) completed the examination, and the changes were present at birth in 112 (22.17%) newborns. These newborns were referred for diagnostic BAEP, and the hearing loss was confirmed in 16 cases.

Hence, 112 (1.43%) of the 7,800 participants in the program were referred for diagnosis. Three (0.03%) children who had been submitted to NHS without RIHL at birth passed the TEOAE but had a delayed speech and language development, observed in the child development monitoring in primary health care. Thus, 115 (1.47%) children were referred for this stage, 19 of whom were referred to the high-complexity service to confirm the diagnosis and, if necessary, receive the hearing aid.

The mean age at the first TEOAE screening was 17 days. Of the total sample, 91% were submitted to it by 30 days old; the minimum age was 2 days, and the maximum age, 713 days.

The results of the diagnosis and rehabilitation stages are described in Table 2. Regarding the diagnosis, 19 (0.24%) of the 7,800 participants were referred to the Regional Hospital of Divinolândia – Conderg, 18 (94.73%) of whom attended it.

**Table 2:** Description of the quality indicator regarding the diagnosis and rehabilitation stages

**Variable**	**Frequency**	**Percentage**
**Referral for diagnosis at the high-complexity service**		
**Yes**	19	0.24
**No**	7.800	99.76
**Attendance at the high-complexity service**		
**Yes**	18	94.73
**No**	1	5.77
**Diagnosis confirmed by three months old at the high-complexity service**		
**Yes**	**3**	**16.66**
**No**	**18**	**83.34**
**Confirmed diagnosis of hearing loss**		
**Permanent hearing loss – sensorineural**	**12**	**66.66**
**Suggestive of auditory neuropathy**	**2**	**16.66**
**Transitory hearing loss – conductive**	**5**	**27.79**
**Normal Hearing**	**1**	**5.55**
**Candidates to hearing aid who received within one month from diagnosis**		
**Yes**	**1**	**10.00**
**No**	**9**	**90.00**
**Participants with permanent bilateral or unilateral hearing loss who began speech-language-hearing therapy immediately after the diagnosis was confirmed**		
**Yes**	**12**	**100**
**No**	**0**	**0**

Source: The researcher's own database.

Three (16,66%) of the 18 participants who attended the diagnostic assessment had it confirmed by 3 months old. The mean age at confirmed diagnosis was 21 months, and the maximum age, 6 years.

One (5.55%) of the 18 infants who attended the diagnostic assessment was diagnosed as normal-hearing, 12 (66.66%) were diagnosed with sensorineural hearing loss – two of them (16.66%) suggestive of auditory neuropathy –, and five (27.79%) were diagnosed with conductive hearing loss. Therefore, the prevalence of permanent hearing loss in the period of the study was 1.53 per 1,000 live births, and that of transitory hearing loss was 0.06 per 1,000 live births.

As for the rehabilitation stage, 10 participants were candidates for hearing aids, 2 (20%) of whom received the device within 1 month from diagnosis.

The 12 children diagnosed with permanent hearing loss began the speech-language-hearing therapy immediately after confirming the diagnosis. The first instructions on issues related to deafness were given before they received the hearing aids.

## DISCUSSION

The sensitivity of children’s first NHS must be as close to 100% as possible, and it must be performed while they are still newborn – which is why it is called neonatal. The program in question is carried out at a municipal health service belonging to the Unified Health System and therefore follows the principles of comprehensiveness, universality, and equity. Thus, this service attends children whose first visit to a doctor takes place after the neonatal period. In this study, we included children who came to the service after 1 month old and those who were more than 2 years old, either living in the municipality or coming from another one and who had never been submitted to an NHS. Although their data might compromise the measures, we chose to maintain them in this analysis to describe the quality of the service and portray the reality we meant to assess.

The analysis of the results reveals that the program met five out of the seven indicators assessed in this paper. Hence, it is feasible to conduct a child hearing health program in an outpatient setting.

The first stage in such programs is the NHS with TEOAE. The quality indicator for this stage requires at least 95% coverage of newborns, aiming at 100%, which qualifies it as universal NHS. Moreover, this stage must be concluded within the newborn’s first month of life^(2)^.

This study results showed that the outpatient program met the indicators for the first stage, as established by the NHS Care Guidelines^(2)^.

The coverage rate in the present study was higher than the one presented in two national papers carried out in 2014 and 2016 – the results in the first of them show a national coverage of 7.1% in 2008 and 21.8% in 2011^(12)^. In the second piece of research, the national coverage was 37.2%^(12)^. After the federal law that made the NHS mandatory, this indicator has increased, although it is still short of the expected and cannot be considered universal.

These two national studies also pointed out the possibility of underreported and overestimated values, due to the use of secondary data from the information system of the Ministry of Health. One of the factors for underreporting is the outdated data in the national information system, while the overestimated values are explained by the single procedure number used for tests and retest^(12,13)^.

It is important to highlight that this research likewise used secondary data. However, they proved to be more trustworthy than the ones used in the previous research in 2014 and 2016, as they were updated in the SINASC monthly spreadsheets sent by the Municipal Epidemiological Surveillance to the program coordinators. The data in this spreadsheet also made it possible to verify the newborns who did not live in the municipality and thus refer them to their city of origin, with feedback from the SINASC. Hence, they were not included in the coverage calculation.

A literature review on the coverage of the hearing health programs in Brazil showed that most of them were conducted in public maternity hospitals and less than half met the universal coverage. The factors that negatively influenced these results were the early hospital discharge and the few speech-language-hearing therapists hired to make examinations on all the days of the week^(14)^. Nevertheless, such factors do not corroborate the present study, as the program conducted in an outpatient setting managed to screen 97% of the newborns.

The following factors contributed to reaching the universal coverage in the present study: the possibility of hiring a speech-language-hearing therapist for 10 hours a week, which is necessary to test and retest approximately 100 live newborns a month; the unified database; the constant interface with the other parts of the municipal and intermunicipal health network; and the constant active search for those who did not attend the screening.

An important point is having universal and free access to the program in the municipality, following the principles of the Unified Health System. This means that everyone has the right to all the stages of the child hearing health program, which also helps reach universal coverage. Similar universal coverage data were found in a maternity hospital in the municipality of Marília, São Paulo, likewise conducted in an outpatient setting, whose coverage was 96.3%. A determining factor to reach this quality indicator, according to the researchers, was that it was a low-risk maternity^(5)^.

Hearing health programs must be constantly analyzed to improve the quality indicators. This was verified in the study that addressed a program in Várzea Grande, Mato Grosso, which was initially carried out at hospitals but then was transferred to outpatient centers. This revealed a 90% coverage, as the data were entered into a database with this very purpose^(15)^.

Most of the 7,800 participants passed the first stage of the test, and a high percentage of those who failed attended the retest. These data are similar to those in other studies^(6,16)^.

Regarding the age at the first NHS, it can be stated that the program met the quality indicator – i.e., 91% of the participants were submitted to the first TEOAE screening by 30 days old. Similar mean age data were found in other pieces of research, also carried out in outpatient settings, such as the one from the Center for Studies and Research in Rehabilitation (Cepre, in Portuguese) in Campinas, from Belo Horizonte, and from Várzea Grande, with a mean age of 24, 23, and 30 days^(17,14,16)^. In another research carried out in Belo Horizonte, also in an outpatient setting, approximately 65.1% were screened by the first month of life^(6)^.

Although the mean age at the first TEOAE screening was 17 days, some children were submitted to it after 1 month old. This happened for different reasons, such as the neonatal ICU stay, nonattendance on the day of the examination, and children born in other municipalities. Nonetheless, the program administration, based on the SINASC spreadsheet, is always actively searching for the children who did not attend or were not included in the first screening. The same procedure is used with the children who first come to the outpatient center after 1 month old – i.e., performing the TEOAE screening as the first test.

After the first stage with the TEOAE screening and the second one with the retest, the diagnosis makes up the third stage in the child hearing health programs. It is considered a quality program when 2% to 4% of the participants are referred for this stage, with 90% attendance of those referred, and the diagnosis confirmed by 3 months old^(2)^. The diagnosis stage results in this research showed that the program met the indicator in terms of the percentage of attendance (with 94.73% of the participants) and the 0.24% rate of participants referred for diagnosis, in comparison with the literature^(2)^. On the other hand, the expected percentage of age at confirmed diagnosis (by 3 months old) did not meet the quality indicators recommended in the literature^(2)^.

Other studies also met the indicator of the percentage of referrals for diagnosis, with 0.3%, 2.1%, and 0.9%^(6,7,18)^. On the other hand, the data are different from research conducted in Batatais, with 6.02% of the participants referred for diagnosis^(16)^.

In the municipality, 112 participants who continued with a failed result were referred for diagnostic BAEP examination, after having performed this same test in the screening.

The municipality has this equipment available, which may be a facilitator in this stage. In many cases, the diagnosis is reached late, as shown in the study carried out in Batatais, which refers all newborns with RIHL or who fail the TEAOE to the hearing health reference service in another city because they do not have the equipment^(16)^.

Regarding the attendance to the high-complexity service, the results in the present study are similar to research conducted at a low-risk maternity hospital in the state of São Paulo, with 99% attendance^(5)^. They were different, though, from national and international studies, which did not reach the diagnosis attendance rate recommended in the literature^(16,19)^.

Some obstacles were described in a study that did not reach the diagnosis adherence rate, namely: the decentralized regulation of the NHS program, the little availability for scheduling a diagnostic assessment in the high-complexity service, the difficulties with public transportation, and the distance to the other municipality. Consequently, these factors help increase the percentage of participants lost before concluding this stage^16.^

Nonetheless, the factors pointed out in the said research, which negatively influenced the adherence to diagnosis, were not observed in the present study. Besides the constant active search, the child hearing health program regulation, in this case, is centralized in the same service, the reference for diagnosis gives priority to children, and the municipality provides the necessary intermunicipal transportation. In another study carried out in four hospitals in Thailand, the adherence to diagnosis was 78%, but when they began contacting the families a few days before the examination, they increased the percentage of attendance, making it a facilitating action towards adherence in this stage^(19)^.

As for the age at confirmed diagnosis, the quality indicator in the literature recommends that this stage be concluded by 3 months old^(2)^. The data found in the present research did not meet the recommendations, as only three (13.66%) of the participants had this stage concluded by the third month of life.

Other studies also showed similar data concerning the age at confirmed diagnosis. One of them, carried out in Teresina, showed the programs’ difficulty confirming the diagnosis at the expected age, with results ranging from 7 months to 1 year old^(20)^. This situation was also verified in another study at the *Universidade do Vale do Itajaí* (University of the Itajaí Valley), as 15.38% of the participants had their diagnosis confirmed by 3 months old, and 34.61%, after 24 months old^(21)^. Also, research from São Paulo found that 18.4% of the children had been diagnosed at the age recommended in the literature^(22)^. However, in research from Curitiba, the program met the rate of confirmed diagnosis by 3 months old as recommended by the NHS Care Guidelines^(12)^.

The sensorineural hearing loss was the most frequent result among the 18 infants who attended the diagnosis – two of which had a suspicion of auditory neuropathy spectrum disorder. These data are similar to the ones found in the studies of hearing health services of Batatais and the north coast of Santa Catarina^(16,21)^.

Concerning transitory conductive hearing loss, a lower percentage (0.59%) was also found in a study from a low-risk maternity hospital in inland São Paulo. However, it did not find any hearing loss suggestive of auditory neuropathy spectrum disorder, as most of the babies assessed did not have RIHL^(5)^. Contrarily, another research, with a higher rate of participants with RIHL, found 0.2% with suspicion of auditory neuropathy^(23)^. Therefore, regarding the type, a higher percentage of sensorineural hearing loss was found in other studies, as in the present research.

A 1.53 prevalence of permanent hearing loss per 1,000 live births and a 0.06 prevalence of transitory hearing loss per 1,000 live births were found in the period of the study. According to the literature, the prevalence of permanent hearing loss ranges from one to six newborns per 1,000 live births^(2)^. National studies report a 0.9% prevalence of permanent hearing changes, regardless of RIHL^(24,25)^.

The 1.53 prevalence per 1,000 live births is a lower result than that of a high-risk maternity hospital in Campinas, with five cases of hearing loss per 1,000 live births^(16)^. However, that is a reference maternity hospital for mothers and babies at risk – a different situation from the other pieces of research, which encompassed newborns from the whole city, not only those at risk. The differences in population and methodology between the hearing health programs may explain the various prevalence results in Brazilian studies^(5)^.

The last quality indicator analyzed was the hearing aid fitting, which must be provided to 95% of the babies within 1 month from diagnosis. In this study, one (10%) patient met this indicator, which is below the rate recommended in the literature. Similar results were found in other studies^(24,26)^. It is worth pointing out that even in the research from Curitiba, with diagnoses within 3 months of life, they received the hearing aid at 6 months old^(27)^.

A study conducted at a hospital in Chile also had difficulties fitting the hearing aid within 1 month from diagnosis; instead, the time to receive it was 4.4 months. This delay may be explained by the need for government procurements to acquire equipment and by one participant’s family’s likely denial of the diagnosis of hearing loss^(25)^. The Regional Hospital of Divinolândia – Conderg, which provides reference high-complexity attention to patients from Mogi Mirim, faces the same difficulty regarding the slow government procurement process to acquire hearing aids, besides the limited number of hearing aids made available and distributed in each municipality.

The literature recommends, as a quality indicator, that the speech-language-hearing therapy begin immediately after the diagnosis in 95% of children with permanent bilateral or unilateral hearing loss^(2)^. The study results revealed that the program reached this quality goal, as all families received the first instructions about deafness issues after finishing the examinations in the municipality. Thus, they obtained rehabilitation with the multiprofessional team – i.e., with the speech-language-hearing therapist, psychologist, and social worker.

Other studies also had difficulties reaching a diagnosis by 3 months old, with the therapy beginning at 7 months to 1 year old, on average, or even after 4 years old^(20,28)^. This is a rather important phase, as studies indicate that, when the hearing loss is diagnosed by 3 months old and the therapy intervention starts by 6 months old, the language development may be compatible with that of same-age normal-hearing children^(28)^. These studies show that the hearing health programs have a great challenge regarding the age at confirmed diagnosis and immediate rehabilitation. Also, the programs need to rethink the work process to obtain better rates in the diagnosis stage and, consequently, in the development of children diagnosed with hearing loss.

It is important to highlight that, although the present study did not have the diagnoses confirmed by 3 months old, the rehabilitation was ensured after finishing all the stages in the program.

Another point to emphasize is the scarcity of national studies addressing the diagnosis stage, hearing aid fitting, and beginning of rehabilitation. This is one of the reasons to reconsider the lack of a municipal information system, which compromises the assessment of all quality indicators of a hearing health program, the planning and referral to child health care, the patients’ comprehensive health care, and the allocation of financial resources^(14,24)^.

A limitation in our study was the difficulty diagnosing children by 3 months old, as recommended in the national and international literature, besides the participants lost in the BAEP screening stage.

Some factors can explain this limitation. For instance, the BAEP in the screening stage, used in participants with RIHL, was not performed on the same day of the first TEOAE screening; the retest was conducted with TEOAE instead of BAEP in screening mode, as instructed in the literature^(2)^; when the BAEP equipment needs repair, the process takes too long; the middle ear infections that delay the diagnostic process; the relatives’ nonattendance to the examinations.

Another limitation is the age at hearing aid fitting because it depends on the Regional Hospital of Divinolândia – Conderg. This situation can be improved with the annual health pact, approved by the Municipal Health Council for 2021, to implement a Specialized Rehabilitation Center in the city this year.

The analysis of all the stages of the program was only made possible thanks to the constant communication between the municipal hearing health regulation and the hearing health high-complexity reference Reginal Hospital of Divinolândia – Conderg, besides the other health services in the municipal and intermunicipal network, the constant active search, the database implemented together with the program, and the information entered into it.

Other important indicators to be considered refer to writing the results in the personal child health record, the constant active search on the part of the hearing health regulation, and the primary health care administrators and professionals – especially the community health agents, as they are always attentive to verify, in the personal child health records, whether the newborns were submitted to the examinations.

## CONCLUSION

The NHS program of the municipality of Mogi Mirim, implemented in an outpatient setting, met the quality indicators described in the literature regarding its coverage, age at the NHS, beginning of the speech-language-hearing therapy, percentage of referrals for diagnosis, and rate of attendance to diagnosis. However, a challenge remains, which is to lower the age at confirmed diagnosis and hearing aid fitting.

It can be stated that the program’s quality goals were achieved, thanks to the dedication of the professionals of the service, involving the teamwork process to implement the program, the cooperation and commitment of all professionals in the municipal health network, the hearing health service regulation, and the administration of the other health facilities in this and other cities, to refer the newborn for screening and constant active search.
