# Supplementary material for: Analysis of an outpatient child hearing health program: from screening to referral for rehabilitation
Source: Codas. 2022 Feb 2;34(4):e20200403. doi: 10.1590/2317-1782/20212020403 (PMC9886126; doi:10.1590/2317-1782/20212020403)
Supplement: Translation

# Translation: Análise de um programa de saúde auditiva infantil ambulatorial: da triagem ao encaminhamento para reabilitação

**Authors:** Kátia de Cássia Botasso, Maria Cecília Marconi Pinheiro Lima, Carlos Roberto Silveira Correa

**Affiliations:** 1 Departamento de Saúde Coletiva, Faculdade de Ciências Médicas, Universidade Estadual de Campinas – UNICAMP - Campinas (SP), Brasil.; 2 Departamento de Desenvolvimento Humano e Reabilitação, Faculdade de Ciências Médicas, Universidade Estadual de Campinas – UNICAMP - Campinas (SP), Brasil.

**Keywords:** Triagem Neonatal, Avaliação de Programas e Projetos de Saúde, Perda Auditiva, Diagnóstico, Reabilitação

## Abstract

**Objetivo:**

Analisar as etapas de um programa de saúde auditiva, da triagem ao encaminhamento para reabilitação, segundo os indicadores de qualidade de programas de triagem neonatal.

**Método:**

Trata-se de um estudo de coorte, observacional e retrospectivo, constituído por todos os neonatos inscritos no Sistema de Informação Municipal de Mogi Mirim/SP, de 2010 a 2016. Além dos dados que constam no Sistema de Informações sobre Nascidos Vivos, foram analisados idade do neonato no primeiro teste, resultado dos testes, do diagnóstico e encaminhamento para reabilitação. A análise dos dados foi feita segundo os critérios de indicadores de qualidade das diretrizes de atenção à triagem auditiva neonatal, por meio de programa estatístico.

**Resultados:**

Participaram 7.800 neonatos e com relação à análise dos indicadores de qualidade do programa foram obtidos os seguintes resultados: 1) Etapa da TAN: 97% de cobertura do primeiro teste; 91% dos neonatos com até 30 dias de vida; 2) Etapa do Diagnóstico: 0,24% encaminhados após falharem no segundo teste; 94,73% de adesão; 13,66% concluíram até os três meses de idade e 3) Etapa da Reabilitação: 100% iniciaram terapia fonoaudiológica imediatamente após o diagnóstico; 20% receberam o aparelho de amplificação sonora individual com até um mês do diagnóstico.

**Conclusão:**

O programa, realizado em nível ambulatorial, atingiu as recomendações das Diretrizes do Ministério da Saúde com relação à cobertura e idade do primeiro exame, idade da triagem até um mês de vida, encaminhamento para diagnóstico e início da intervenção. Tais resultados só puderam ser obtidos com o apoio institucional do município.

## INTRODUÇÃO

Programas de saúde auditiva infantil são implantados para se detectar a perda auditiva em tempo oportuno, e assim minimizar as dificuldades decorrentes da privação sensorial. Para o alcance desse objetivo tanto o *Joint Committee on Infant Hearing* (JCIH) como as Diretrizes de Atenção da Triagem Auditiva Neonatal do Ministério da Saúde propõem cinco etapas aos programas de saúde auditiva: teste, reteste, diagnóstico, reabilitação e monitoramento e acompanhamento do desenvolvimento da audição e linguagem^(1,2)^.

A lei Federal nº 12.303, de dois de agosto de 2010, tornou obrigatório e gratuito o rastreamento para a detecção precoce da perda auditiva com a realização do exame de Emissões Otoacústicas Evocadas em ambiente hospitalar^(3)^.

No município de Mogi Mirim, as discussões a respeito da importância da implantação de um programa de saúde auditiva ocorreram em 2005. Nesse ano, a coordenação de saúde apresentou um projeto para esse programa aos gestores municipais, proposta que foi aprovada no ano seguinte durante a conferência municipal de saúde. Em seguida, foi promulgada a lei municipal nº 12.522, de dois de janeiro de 2007, sobre a obrigatoriedade de se realizar o exame de Emissões Otoacústicas em crianças do município, em ambiente ambulatorial^(4)^, mas a implantação do Programa de saúde auditiva infantil ocorreu em 2009, após a gestão municipal aprovar a aquisição dos equipamentos e a formação da equipe de triagem e da reabilitação. Portanto, o controle social e a interface com o poder legislativo foram fundamentais para a implantação do programa de saúde auditiva infantil no município.

O Programa de Saúde Auditiva Infantil teve início na Atenção Primária à Saúde, com orientações para as gestantes, implementadas pela equipe multiprofissional, composta pelas áreas de enfermagem, fonoaudiologia, ginecologia, psicologia, nutrição e serviço social. O profissional fonoaudiólogo orienta a respeito da importância do aleitamento materno, das consequências dos hábitos deletérios, da importância das etapas do programa de saúde auditiva: teste e reteste, diagnóstico, reabilitação (terapia fonoaudiológica e concessão da prótese auditiva) e a respeito do monitoramento e acompanhamento do desenvolvimento da audição e linguagem da nossa população infantil, na Atenção Primária (AP).

O Programa realiza as etapas de teste, reteste, reabilitação (terapia fonoaudiológica) e monitoramento do desenvolvimento, no próprio município do estudo, mas quando é necessário o diagnóstico e a protetização, encaminha-se os recém-nascidos (RN) ao Hospital Regional de Divinolândia - Conderg, situado no município de Divinolândia/SP, referência em saúde auditiva.

Para se avaliar um programa de saúde infantil, de acordo com os indicadores de qualidade propostos pelas Diretrizes de Atenção da Triagem Auditiva Neonatal do Ministério da Saúde: 1) Etapa da triagem auditiva neonatal: cobertura da TAN em 95% e com objetivo de alcançar 100%, idade de até um mês no momento da TAN e 2% a 4% de encaminhamento ao diagnóstico; 2) Etapa do diagnóstico: no mínimo, 90% de comparecimento e conclusão dessa etapa até três meses de idade; 3) Etapa da reabilitação: 95% dos neonatos com início de terapia fonoaudiológica, após a conclusão do diagnóstico; e 95% de neonatos com perdas auditivas bilaterais ou unilaterais permanentes devem receber aparelho de amplificação sonora individual, imediatamente após a confirmação de perda auditiva^(2)^.

Vários estudos nacionais analisam indicadores de qualidade de programas de triagem auditiva neonatal, porém, em geral, com a utilização de bancos de dados de uma ou mais maternidades e não da população compreendida por todos os nascidos vivos de um município. Além disso, são encontradas pesquisas de alguns indicadores propostos pelas Diretrizes de Atenção da Triagem Auditiva Neonatal, tais como a idade da realização do primeiro exame da TAN e o índice de encaminhamento ao diagnóstico^(5-7)^.

O presente estudo justifica-se pela descrição de todas as etapas e dos indicadores de qualidade de um Programa de Saúde Auditiva Infantil, e poderá ser modelo para outras equipes que pretendem iniciar projetos desta natureza.

Diante do exposto, o objetivo desse estudo foi analisar todas as etapas de um programa de saúde auditiva, desde a triagem até o início da reabilitação, segundo os critérios dos indicadores de qualidade de programas de triagem neonatal.

## MÉTODO

O projeto de pesquisa foi submetido ao Comitê de Ética em Pesquisa da Universidade Estadual de Campinas (UNICAMP), com aprovação dia 06/02/2018, sob o nº de parecer 2.487.739. Entretanto, para esse estudo, não foi utilizado o Termo de Consentimento Livre e Esclarecido, por se tratar de dados secundários, pois foram coletados, ordenados e tabulados previamente, sendo que muitas informações são de domínio público^(8)^.

Trata-se do estudo de uma coorte, observacional e retrospectiva, constituída por todos os 7.800 participantes inscritos no Sistema de Informação Municipal, e que participaram do programa de saúde auditiva infantil do município de Mogi Mirim, no período de 2010 a 2016.

A primeira etapa do Programa se caracteriza com a realização do exame de EOAT, que se tornou obrigatória em 2007, em nível ambulatorial, no Centro de Especialidades de Mogi Mirim, local em que os Fonoaudiólogos do município exercem suas atividades. Entretanto, a implantação do programa de saúde auditiva ocorreu em 2009, após a gestão municipal aprovar a aquisição dos equipamentos e formação da equipe de triagem e reabilitação.

Para essa pesquisa, utilizou-se dados secundários, assim denominados, pois foram coletados, ordenados e tabulados previamente, sendo que muitas das informações são do domínio público^(8)^. Esses dados foram obtidos de um livro de registro, implantado desde o início do programa de saúde auditiva, sendo considerado o primeiro banco de dados do programa.

Nesse primeiro banco de dados, foram inseridas as informações dos participantes residentes em Mogi Mirim, que realizam o teste de EOAT no Ambulatório de Fonoaudiologia, situado no Centro de Especialidades de Mogi Mirim (CEMM) ou em outras Instituições contidas no Sistema de Informação sobre Nascidos Vivos (SINASC), além das variáveis sociodemográficas da família; história clínica com o indicador de risco para deficiência auditiva (IRDA); data e resultados dos exames realizados na fase de teste, reteste e diagnóstico, encaminhamentos realizados; data da entrega da prótese auditiva e início da terapia fonoaudiológica.

A partir do ano de 2015, o registro dessas informações foi inserido em uma planilha eletrônica, para facilitar a avaliação dos indicadores de qualidade do programa, e consequentemente, possibilitou a ampliação de dados referentes ao acompanhamento do período gestacional das genitoras dos participantes e do programa de monitoramento do desenvolvimento da audição e da linguagem.

O teste de EOAT foi realizado com o equipamento Maico Ero-Scan, com o teste das frequências de 1,5 kHz a 4 kHz, sendo considerado como critério de passa, a relação sinal/ruído superior a 4 dBNPS em 1 kHz e superior a 6 dBNPS nas demais frequências, em pelo menos 3 frequências avaliadas, incluindo 4 kHz, além da observação do Reflexo Cócleo-palpebral, com o instrumento agogô. Considerou-se que o neonato “passou” no teste e reteste, ou seja, sem alteração auditiva, quando o reflexo cócleo-palpebral (RCP) e as EOAT estavam presentes em ambas as orelhas. Porém, quando as respostas auditivas não estavam presentes, denominou-se que o neonato “falhou” na TAN.

Todos os neonatos que falharam no primeiro teste foram encaminhados à fase de reteste e quando a falha persistiu, realizaram-se os exames de medidas de imitanciometria, ou seja, a timpanometria e a pesquisa do reflexo acústico. Quando os resultados de medidas de imitância não estavam dentro da normalidade, segundo o guia de orientação da avaliação audiológica do sistema de conselhos de fonoaudiologia^(9)^, os participantes eram encaminhados ao médico otorrinolaringologista e as EOAT eram refeitas.

Entretanto, quando o resultado do exame das medidas de imitância era classificado como normal (curva timpanométrica do tipo A e reflexo acústico presente, mas o neonato falhava nas EOAT, era encaminhado para o exame de Potencial Evocado Auditivo de Tronco Cerebral (PEATE), com o equipamento Pentetek – Audtec – Potencial de tronco cerebral do sistema Audiscan que avalia, por meio do estímulo clique, a integridade da via auditiva e as frequências de 2 a 4 kHz, e com frequência específica, a de 1000Hz.

O neonato foi considerado com IRDA, na presença de pelo menos um dos seguintes indicadores sugeridos pelo *Joint Committee on Infant Hearing* ^(1)^ e pelas Diretrizes de Atenção da Triagem Auditiva Neonatal^(2)^: preocupação dos pais com o desenvolvimento da criança, da audição, fala ou linguagem; antecedente familiar de surdez permanente e consanguinidade; permanência na UTI por mais de cinco dias; ventilação mecânica; exposição a drogas ototóxicas; anóxia perinatal grave; Apgar neonatal de 0 a 4 no primeiro minuto, ou 0 a 6 no quinto minuto; peso ao nascer inferior a 1.500 gramas; infecções congênitas (toxoplasmose, rubéola, citomegalovírus, herpes, sífilis, HIV); anomalias craniofaciais envolvendo orelha e osso temporal; síndromes genéticas; hiperbilirrubinemia; distúrbios neurodegenerativos; infecções bacterianas ou virais pós-natais (citomegalovírus, herpes, sarampo, varicela e meningite); traumatismo craniano; quimioterapia^(1,2)^. Foram incluídos também, alcoolismo materno ou uso de substâncias psicotrópicas na gestação, hemorragia peri-intraventricular e convulsões neonatais^(10)^. Importante destacar que os indicadores de risco utilizados para esse estudo são referentes à declaração de posicionamento do JCIH do ano de 2007^(1)^, pois esta só foi reeditada em 2019^(11)^, momento em que a pesquisa já estava finalizada.

Quando constatada alteração nos exames, encaminhou-se ao Hospital Regional de Divinolândia - Conderg, para a confirmação do diagnóstico, realização de exames complementares e protetização. Em seguida, a família recebeu as primeiras orientações, no próprio município, quanto à reabilitação. Por último, todos os participantes foram encaminhados ao programa de monitoramento e observação do desenvolvimento infantil, na Atenção Primária à Saúde, com a Fonoaudióloga de referência. Essa é uma prática que existe no município há muitos anos, em que todas as crianças são acompanhadas quanto ao seu desenvolvimento de forma global, inclusive o desenvolvimento auditivo, em função das perdas progressivas ou de início tardio.

Para analisar os indicadores da primeira etapa do Programa, foram consideradas as variáveis: Cobertura da triagem, idade em dias em que realizou o primeiro teste e resultados dos exames no teste e reteste, média de idade para diagnóstico. Na fase do diagnóstico, foram analisadas as variáveis: Encaminhamento ao diagnóstico, comparecimento na alta complexidade, idade do lactente na finalização do diagnóstico, conclusão do diagnóstico, tipos de perdas e prevalência de perdas permanentes e temporárias. Na fase de reabilitação, as variáveis foram: Encaminhados para prótese auditiva; idade em que receberam as próteses; idade em que iniciaram a terapia fonoaudiológica.

Utilizaram-se os parâmetros sugeridos pelas Diretrizes de Atenção da Triagem Auditiva Neonatal, para a análise dos indicadores de qualidade do programa^(2)^:

1. Etapa da TAN: cobertura de 95% referentes aos nascidos vivos declarados residentes, idade em que realizou o teste até 30 dias de vida.
2. Etapa do diagnóstico: índice entre 2% e 4% de encaminhamentos ao diagnóstico (para alta complexidade); comparecimento na alta complexidade de 90%; e conclusão do diagnóstico até três meses de idade.
3. Etapa de reabilitação: 95% dos candidatos devem receber prótese auditiva, após um mês do diagnóstico; 95% dos lactentes confirmados com perdas auditivas bilaterais ou unilaterais permanentes, deverão ter o início de terapia fonoaudiológica imediatamente após a conclusão do diagnóstico.

Realizou-se análise descritiva da distribuição de frequência das variáveis categóricas, e valores percentuais. Porém, para a cobertura também se utilizou da média e desvio padrão e para a variável idade da realização do primeiro exame de EOAT, utilizaram-se as medidas do primeiro, segundo e terceiro quartil. E todos os dados foram analisados pelo programa estatístico SAS.

Por último, utilizou-se a medida da prevalência para as perdas auditivas permanentes e temporárias, sendo considerada: 1) número de casos com perdas auditivas permanentes, durante o período do estudo, sob o número total de participantes, durante o período do estudo; 2) número de casos com perdas auditivas temporárias, durante o período do estudo, sob o número total de participantes, durante o período do estudo.

## RESULTADOS

Analisaram-se as informações referentes aos 7.800 participantes do programa de saúde auditiva infantil no período de 2010 a 2016.

Na Tabela 1, demonstram-se os dados quanto à cobertura do primeiro teste de EOAT dos recém-nascidos residentes, sendo observado que no primeiro ano (2010) a cobertura foi de 93%, mas com média do período do estudo de 97,00% (DP: 0,02).

**Tabela 1:** Descrição do Indicador de qualidade referente à cobertura do primeiro exame de EOA em nascidos vivos residentes no município

Ano de Nascimento	Nascidos Vivos Residentes	Frequência	Porcentagem
	N= 7.962	N= 7.800	%
2010	1144	1072	93,00
2011	1089	1075	98,00
2012	1153	1142	99,00
2013	1168	1123	96,00
2014	1154	1120	97,00
2015	1157	1151	99,00
2016	1097	1043	95,00
Média: 97,00; Desvio Padrão: 0,02			

Fonte: Banco de dados da própria pesquisadora.

Referente à primeira etapa do teste, dos 7.800 participantes, 7.294 (93,51%) passaram e 506 (6,49%) falharam, sendo encaminhados ao reteste.

Dos participantes que foram encaminhados ao reteste, 460 (90,90%) compareceram, 348 (75,65%) passaram e 112 (24,35%) continuaram com o resultado de falha.

Encaminhou-se ao PEATE, como triagem, 1049 (13,48%), sendo que 937 (89,32%) foi devido à presença de IRDA ao nascimento, 56 (5,34%) falharam no reteste do EOAT e apresentaram IRDA ao nascimento, e 56 (5,34%) falharam no reteste do EOAT e não tinham IRDA ao nascimento. Compareceram 738 (70,48%), e destes, 505 (48,23%) completaram o exame, sendo que em 112 (22,17%) neonatos, as alterações estavam presentes ao nascimento. Esses neonatos foram encaminhados ao PEATE de diagnóstico, e em 16 casos, houve a confirmação da perda auditiva.

Entretanto, para o diagnóstico, dos 7.800 participantes do programa, encaminhou-se 112 (1,43%) neonatos. Três (0,03%) crianças que realizaram a triagem auditiva, sem IRDA ao nascimento, passaram na EOAT, mas apresentaram atraso no desenvolvimento da fala e da linguagem durante o monitoramento do desenvolvimento infantil, na Atenção Primária à Saúde. Sendo assim, foram realizados 115 (1,47%) encaminhamentos para essa fase, e destes, 19 foram encaminhados para a Alta Complexidade, para a confirmação do diagnóstico e se fosse necessário, a protetização.

Com relação à idade da realização do primeiro exame de EOAT, a média de idade foi de 17 dias, 91% do total de participantes realizou até os 30 dias, sendo a idade mínima dois dias e a máxima de 713 dias.

Na Tabela 2, foram descritos os resultados da fase do diagnóstico e reabilitação. Quanto à fase do diagnóstico, dos 7.800 participantes, 19 (0,24%) foram encaminhados para o Hospital Regional de Divinolândia – Conderg, com 18 (94,73%) comparecimentos.

**Tabela 2:** Descrição dos Indicadores de qualidade referente à fase do diagnóstico e reabilitação

**Variável**	**Frequência**	**Porcentagem**
**Encaminhamento ao diagnóstico na alta complexidade**		
**Sim**	19	0,24
**Não**	7.800	99,76
**Comparecimento na alta complexidade**		
**Sim**	18	94,73
**Não**	1	5,77
**Conclusão do diagnóstico até três meses de idade na alta complexidade**		
**Sim**	**3**	**16,66**
**Não**	**18**	**83,34**
**Conclusão do diagnóstico das Perdas auditivas**		
**Perda auditiva permanente - neurossensorial**	**12**	**66,66**
**Sugestiva de neuropatia auditiva**	**2**	**16,66**
**Perda auditiva transitória – condutiva**	**5**	**27,79**
**Audição Normal**	**1**	**5,55**
**Candidatos à prótese e receberam, após um mês do diagnóstico**		
**Sim**	**1**	**10,00**
**Não**	**9**	**90,00**
**Participantes com perda auditiva bilateral ou unilateral permanente, iniciaram terapia fonoaudiológica imediatamente, após a conclusão do diagnóstico**		
**Sim**	**12**	**100**
**Não**	**0**	**0**

Fonte: Banco de dados da própria pesquisadora.

Referente à idade da conclusão do diagnóstico, dos 18 que compareceram, três (16,66%) participantes concluíram até os três meses, com média de 21 meses e idade máxima de seis anos.

Dos 18 lactentes que compareceram, um (5,55%) teve o diagnóstico de ouvinte, 12 foram diagnosticados com perda auditiva neurossensorial (66,66%), sendo duas (16,66%) sugestivas de neuropatia auditiva, e cinco (27,79%) com perda auditiva condutiva. Portanto, a prevalência da perda auditiva permanente, no período do estudo, foi de 1,53 em 1.000 nascidos vivos, e da perda auditiva transitória foi de 0,06 em 1.000 nascidos vivos.

Com relação à etapa de reabilitação, 10 participantes foram candidatos à prótese auditiva, sendo que 2 (20%) receberam o equipamento após um mês do diagnóstico.

Quanto ao início da terapia fonoaudiológica, as 12 crianças diagnosticadas com perda auditiva permanente iniciaram essa etapa imediatamente após a conclusão do diagnóstico, com o início das primeiras orientações sobre as questões da surdez antes da protetização.

## DISCUSSÃO

O primeiro exame auditivo de uma criança deve ser, para a triagem de perda auditiva, um teste com sensibilidade o mais próximo de 100%, e ser realizado enquanto ela ainda é recém-nascida. Por isso, o nome Triagem Auditiva Neonatal. O presente Programa, no entanto, está inserido em um serviço municipal de saúde, que atua no Sistema Único de Saúde e que, portanto, trabalha segundo os cânones da integralidade, universalidade e equidade. Dessa forma, esse serviço atende crianças que fazem a primeira consulta em uma idade além do período neonatal. Neste trabalho, incluímos crianças que chegaram ao serviço após um mês de vida e com mais de dois (2) anos, migrantes e/ou residentes, e que nunca haviam passado por uma triagem auditiva. Optamos por deixar na presente análise os dados dessas crianças, mesmo que isso pudesse vir a comprometer as medidas utilizadas, para descrever a qualidade do serviço, em função de ser a realidade que queremos avaliar.

Após a análise dos resultados, pode-se afirmar que o Programa atendeu a 5 dos 7 indicadores que esse trabalho se propôs a avaliar, e dessa maneira, mostra a viabilidade de se realizar um Programa de Saúde Auditiva Infantil de forma ambulatorial.

A primeira etapa de um programa dessa natureza é a triagem auditiva, com o teste de EOAT. Para a análise dos indicadores de qualidade dessa etapa, a cobertura deve ser de pelo menos 95% dos recém-nascidos, com meta de atingir 100%, e dessa maneira ser considerada como TAN universal. Além de atingir o término dessa etapa, até o primeiro mês de vida dos neonatos^(2)^.

No presente estudo, com um programa ambulatorial, verificou-se que os resultados da primeira etapa foram atingidos, quando comparados com os indicadores estabelecidos pelas Diretrizes de Atenção da Triagem Auditiva Neonatal^(2)^.

Ao comparar o índice de cobertura do atual estudo, este foi maior do que o índice apresentado em duas pesquisas nacionais realizadas em 2014 e 2016, pois na primeira os resultados mostraram que a cobertura nacional, em 2008, foi de 7,1% e no ano de 2011, 21,8%^(12)^. Na segunda pesquisa realizada, a cobertura nacional foi de 37,2%^(12)^. Observou-se um aumento desse indicador, após a lei federal, que tornou obrigatória a TAN, mas ainda muito aquém do esperado, para se considerar universal.

Nas duas pesquisas nacionais citadas anteriormente, também se observou a possibilidade de sub-registro e valores superestimados, devido à utilização dos dados secundários dos sistemas de informação do Ministério da Saúde. Um dos fatores ao sub-registro é a desatualização dos dados nos sistemas de informações nacionais, e quanto aos valores superestimados, explica-se pelo fato da utilização de apenas um número de procedimento, para o teste e o reteste^(12,13)^.

Importante ressaltar que, para a atual pesquisa, também foram utilizados dados secundários, entretanto, mostraram-se mais fidedignos, quando comparados às pesquisas anteriores, de 2014 e 2016, pois foram dados atualizados das planilhas mensais do SINASC, enviadas pela Vigilância Epidemiológica do município à coordenação do programa. Além disso, com os dados dessas planilhas, foi possível verificar os recém-nascidos não residentes e dessa maneira encaminhá-los à cidade de origem, por meio da retroalimentação do SINASC, não sendo inserido no cálculo da cobertura.

Um estudo de revisão de literatura sobre a cobertura dos Programas de Saúde Auditiva no Brasil mostrou que a maioria ocorreu em maternidades públicas, e menos da metade conseguiu atingir a cobertura universal, sendo a alta precoce do hospital e o número reduzido de fonoaudiólogos contratados para realizar o exame em todos os dias da semana, os fatores que influenciaram negativamente o alcance desse indicador^(14)^. Entretanto, esses fatores não corroboram com o estudo atual, pois o programa, de forma ambulatorial, conseguiu triar 97% dos neonatos.

Foi possível apontar os seguintes fatores que contribuíram para o alcance da cobertura universal do atual estudo: a possibilidade de dimensionar a carga horária de 10 horas semanais do profissional fonoaudiólogo, necessárias para o teste e reteste, para o atendimento de aproximadamente 100 nascidos vivos mensais; o banco de dados unificado; a constante interface com os demais pontos da rede de saúde municipal e intermunicipal; e a constante busca ativa dos faltosos.

É importante ressaltar que o acesso ao programa no município é universal e gratuito, conforme os princípios do Sistema Único de Saúde, e isso significa que todos possuem o direito a todas as etapas do Programa de Saúde Auditiva infantil, situação que também colabora com o resultado de cobertura universal. Dados semelhantes de cobertura universal foram encontrados em uma maternidade do município de Marília/SP, também em nível ambulatorial, com cobertura de 96,3%. Um fator determinante para atingir esse indicador de qualidade, segundo os pesquisadores, foi a maternidade ser de baixo risco^(5)^.

Interessante descrever a importância da análise constante dos programas de saúde auditiva, com o objetivo de melhorar os indicadores de qualidade, como foi possível verificar no estudo de um programa em Várzea Grande/MT, que inicialmente foi realizado em nível hospitalar, mas posteriormente passou a ser ambulatorial, o que possibilitou o conhecimento da cobertura de 90%, pois os dados foram inseridos em um banco de dados somente para este fim^(15)^.

Referente à primeira etapa do teste, dos 7.800 participantes, a maioria passou e uma grande porcentagem dos que falharam compareceram ao reteste. Esses dados são semelhantes a outros estudos^(6,16)^.

Com relação à idade de realização do primeiro teste da TAN, pode-se afirmar que o programa alcançou o preconizado como indicador de qualidade, ou seja, 91% dos participantes realizaram o primeiro exame de EOAT até os 30 dias de vida. Dados semelhantes, quanto à média de idade, foram encontrados em outras pesquisas, também ambulatoriais, como a do Centro de Estudos e Pesquisa em Reabilitação (Cepre), na cidade de Campinas, do município de Belo Horizonte, e de Várzea Grande (MT), com médias de idade de 24, 23 e 30 dias^(17,14,16)^. Em outra pesquisa, também ambulatorial, realizada em Belo Horizonte, cerca de 65,1% foram triados com até um mês de vida^(6)^.

Embora a média de idade da realização do primeiro exame de EOAT tenha sido de 17 dias, realizou-se o exame de EOAT em crianças após um mês de vida. Isso ocorreu por diferentes motivos, como a permanência em UTI neonatal, falta no dia do exame e nascimentos em outros municípios. Importante descrever que a busca ativa dos faltosos ou das crianças que não realizaram o primeiro exame é realizada constantemente pela gestão do programa, por meio da planilha do SINASC. Com as crianças que comparecem no ambulatório após o primeiro mês de vida, utiliza-se o mesmo procedimento, ou seja, a realização das EOAT como primeiro teste.

Após a primeira etapa com o teste de EOAT e a segunda com o reteste, o diagnóstico compõe a terceira etapa dos programas de saúde auditiva infantil, sendo que se considera como de qualidade, o índice de 2% a 4% dos participantes encaminhados para essa fase, o comparecimento de 90% dos encaminhados e a conclusão do diagnóstico até os três meses de vida^(2)^. Os resultados dessa pesquisa, referente à fase do diagnóstico, mostraram que o programa atingiu o indicador quanto à porcentagem de comparecimento, com 94,73% dos participantes; como também o índice de 0,24% de participantes encaminhados para diagnóstico, quando comparados à literatura^(2)^. Entretanto, a porcentagem da idade de conclusão do diagnóstico esperada, até os três meses de idade, não corroborou com os indicadores de qualidade preconizados pela literatura^(2)^.

No que se refere à porcentagem de encaminhamentos ao diagnóstico, outros estudos também atingiram esse indicador, com 0,3%, 2,1% e 0,9%^(6,7,18)^. Entretanto, os dados diferem da pesquisa realizada em Batatais, com 6,02% dos participantes encaminhados ao diagnóstico^(16)^.

Para o PEATE de diagnóstico, no próprio município, foram encaminhados 112 participantes que continuaram com a falha, após realizarem o mesmo exame, como triagem.

O fato de o município possuir esse equipamento pode ser um facilitador dessa fase, pois em muitos casos, o diagnóstico acontece de modo tardio, como no estudo realizado no município de Batatais, que encaminham todos os recém-nascidos com IRDA ou que falham nas EOAT para o serviço de referência em saúde auditiva em outra cidade, pela ausência do equipamento^(16)^.

Quanto ao comparecimento na alta complexidade, os resultados do atual estudo são semelhantes à pesquisa realizada em uma Maternidade de baixo risco no Estado de São Paulo, com 99% de comparecimento^(5)^. Porém, diferente de outras pesquisas, uma nacional e outra internacional, que não conseguiram alcançar o índice de comparecimento ao diagnóstico preconizado pela literatura^(16,19)^.

Alguns obstáculos foram descritos por um estudo, pois não atingiram o índice de adesão ao diagnóstico, tais como: A regulação descentralizada do programa da TAN, o número restrito de vagas para o agendamento do diagnóstico na alta complexidade, a dificuldade com o transporte municipal e a distância até outro município. Consequentemente, esses fatores colaboraram para uma maior porcentagem de perda de participantes, para a conclusão desta etapa^(16)^.

Entretanto, esses fatores da pesquisa anterior, que influenciaram negativamente a adesão ao diagnóstico, não foram observados no atual estudo, pois a regulação do programa de saúde auditiva infantil é centralizada no próprio serviço, a referência para o diagnóstico prioriza o diagnóstico de crianças, e o município supre a necessidade com relação ao transporte intermunicipal, além da constante busca ativa. Em outro estudo realizado em quatro hospitais da Tailândia, a adesão ao diagnóstico era de 78%, mas quando iniciaram o contato telefônico com os familiares alguns dias antes da data do exame, conseguiram aumentar a porcentagem de comparecimento, sendo uma ação facilitadora à adesão nessa etapa^(19)^.

Quanto à idade da conclusão do diagnóstico, a literatura aponta como indicador de qualidade a realização dessa etapa, até o terceiro mês de vida^(2)^. Os dados encontrados na presente pesquisa não estão em consonância com o preconizado, pois apenas três (13,66%) dos participantes tiveram a conclusão dessa fase, até os três meses de vida.

Outros estudos também mostraram dados similares quanto à idade da conclusão do diagnóstico. Um deles, realizado em Teresina, mostrou a dificuldade que os programas possuem para atingir a idade preconizada na fase de diagnóstico, com resultado entre os sete meses a um ano de idade^(20)^. Essa realidade também foi constatada em outro estudo, na Universidade do Vale do Itajaí, pois 15,38% dos participantes concluíram o diagnóstico com três meses e 34,61%, após os 24 meses de idade^(21)^. Por último, uma pesquisa, em São Paulo, em que 18,4% crianças foram diagnosticadas na idade recomendada pela literatura^(22)^. Entretanto, em uma pesquisa realizada em Curitiba, o programa conseguiu atingir o índice da idade do fechamento do diagnóstico, preconizado pelas Diretrizes de Atenção da Triagem Auditiva Neonatal, até o terceiro mês de vida^(12)^.

Dos 18 lactentes que compareceram ao diagnóstico, a maior frequência foram as perdas auditivas neurossensoriais e destas, duas foram consideradas como suspeitas do espectro da neuropatia auditiva. Esses dados são semelhantes aos encontrados nos estudos de serviços de saúde auditiva, do município de Batatais e do litoral norte catarinense^(16,21)^.

Quanto às perdas auditivas transitórias condutivas, também foi encontrada porcentagem menor (0,59%) no estudo de uma maternidade de baixo risco, no interior de São Paulo. Porém, não se encontrou a presença de perda auditiva sugestiva do espectro da neuropatia auditiva, justificados pela maioria dos bebês avaliados não apresentarem IRDA^(5)^. O que não ocorreu em outras pesquisas, com índice maior de participantes com IRDA, em que encontraram 0,2% com suspeita de neuropatia auditiva^(23)^. Portanto, com relação ao tipo de perda auditiva, observou-se em outros estudos uma maior porcentagem das perdas neurossensoriais, como na atual pesquisa.

Foi encontrada uma prevalência da perda auditiva permanente, no período do estudo, de 1,53 em 1.000 nascidos vivos, e da perda auditiva transitória de 0,06 em 1.000 nascidos vivos. Segundo a literatura, a prevalência da perda auditiva permanente varia de um a seis neonatos para mil nascidos vivos^(2)^. Em estudos nacionais, são relatados prevalência de alterações auditivas permanentes de aproximadamente 0,9%, independentemente da presença de indicador de risco^(24,25)^.

A prevalência de 1,53 casos para cada 1000 nascidos vivos mostra um resultado inferior quando comparado a uma maternidade de risco da cidade de Campinas, com cinco casos de perda auditiva para cada 1000 nascidos vivos^(16)^. Entretanto, a maternidade é referência para risco de saúde da mãe e do neonato, situação diferente das outras pesquisas, que englobaram neonatos de uma cidade como um todo, não apenas os de risco. A diferença entre a população e a metodologia dos Programas de Saúde Auditiva pode explicar os diversos resultados com relação à prevalência nos estudos brasileiros^(5)^.

O último indicador de qualidade analisado foi a adaptação de prótese auditiva, que deve ocorrer em 95% dos bebês, após um mês do diagnóstico. Nesse estudo, um (10%) usuário atingiu esse indicador, um índice inferior ao preconizado pela literatura. Resultados semelhantes foram encontrados em outros estudos^(24,26)^. Interessante ressaltar que mesmo a pesquisa realizada em Curitiba, com o diagnóstico até os três meses de vida, a idade de protetização foi de seis meses^(27)^.

Um estudo realizado em um Hospital do Chile também teve dificuldades em adaptar a prótese auditiva após um mês do diagnóstico, pois a média para o recebimento da prótese auditiva foi de 4,4 meses. Explicações para tal atraso podem estar relacionadas com a necessidade de aquisição de equipamentos, por meio de licitação pública e provável negação do diagnóstico de perda auditiva pela família de um dos participantes^(25)^. O Hospital Regional de Divinolândia - Conderg, referência para a alta complexidade do município de Mogi Mirim, enfrenta a mesma dificuldade referente à morosidade de licitação, para a aquisição das próteses auditivas, além das cotas limitadas de acesso às próteses, distribuídas para cada município.

A literatura recomenda como indicador de qualidade o início de terapia fonoaudiológica imediatamente após o diagnóstico, de 95% das crianças com perda auditiva bilateral e unilateral permanente^(2)^. Os resultados do estudo apontaram que o programa alcançou essa meta de qualidade, pois todas as famílias receberam as primeiras orientações sobre as questões da surdez, após o término dos exames no município, e dessa maneira, tiveram a vaga garantida para a reabilitação com a equipe multiprofissional, ou seja, com os profissionais da fonoaudiologia, psicologia e serviço social.

Outros estudos também encontram dificuldades em realizar o diagnóstico até os três meses de idade, com média de início de terapia, a partir dos sete meses a um ano de idade, ou até mesmo após os quatro anos de idade^(20,2)^. Esta é uma fase muito importante, pois estudos indicam que quando a perda auditiva é diagnosticada até os três meses de vida e a intervenção terapêutica é iniciada até os seis meses, o desenvolvimento da linguagem pode ser compatível com o de crianças ouvintes da mesma faixa etária^(28)^. Esses estudos mostraram que os programas de saúde auditiva possuem um grande desafio quanto à idade da conclusão do diagnóstico e a reabilitação imediata. Além disso, os programas precisam repensar o processo de trabalho, para obterem melhores índices na fase do diagnóstico, e consequentemente para o desenvolvimento das crianças com diagnóstico de perda auditiva.

É importante enfatizar que embora o atual estudo não tenha atingido a conclusão do diagnóstico até os três meses de idade, a reabilitação foi garantida após a finalização de todas as etapas do programa.

Além disso, é importante ressaltar a escassez de estudos nacionais com relação à etapa do diagnóstico, da protetização e do início de reabilitação, sendo uma das razões a ser considerada a ausência de um sistema informatizado municipal, situação que compromete a avaliação de todos os indicadores de qualidade de um programa de saúde auditiva, o planejamento e redirecionamento ao cuidado à saúde infantil, a integralidade aos usuários e alocação de recursos financeiros^(14,24)^.

Uma limitação de nosso trabalho refere-se à dificuldade em se diagnosticar as crianças até os três meses de idade, como preconizado pela literatura nacional e internacional, além da perda de participantes, para o exame PEATE, na fase de triagem.

Alguns fatores podem justificar essa limitação, tais como: A execução do PEATE na fase de triagem, destinado aos participantes com IRDA, não ocorrer no mesmo dia do primeiro exame de EOAT; o reteste ser feito com as EOAT e não com o PEATE em modo triagem, conforme orientações da literatura^(2)^; a morosidade do processo de conserto do equipamento PEATE, quando necessário; as infecções de orelha média que atrasam o processo de diagnóstico e as faltas dos familiares nos exames.

Outra limitação é a idade de protetização, devido à dependência do Hospital Regional de Divinolândia – Conderg, o que poderá ser minimizado com a pactuação anual de saúde, aprovada pelo Conselho Municipal de Saúde, para o ano de 2021, para a implantação de um Centro Especializado em Reabilitação na cidade, ainda nesse ano.

A análise de todas as etapas do programa só foi possível devido à constante comunicação entre a regulação da saúde auditiva municipal com o Hospital Regional de Divinolândia – Conderg, referência da alta complexidade para a Saúde Auditiva, além dos demais pontos de saúde da rede municipal e intermunicipal, a busca ativa constante, a criação do banco de dados desde a implantação do programa e a alimentação das informações.

Outros indicadores importantes a serem considerados relacionam-se à anotação dos resultados na carteira de saúde da criança, a busca ativa realizada constantemente pela regulação da saúde auditiva, a gestão e os profissionais da Atenção Primária, em especial os agentes comunitários de saúde, pois eles estão sempre alertas em verificar, na Caderneta da Criança, se os recém-nascidos realizaram os exames.

## CONCLUSÃO

O programa de triagem auditiva neonatal do município de Mogi Mirim, implantado em nível ambulatorial, atingiu os indicadores de qualidade descritos pela literatura referente à cobertura, idade de realização da TAN, início da terapia fonoaudiológica, porcentagem de encaminhamento ao diagnóstico e índice de comparecimento nessa etapa. Entretanto, ainda com um desafio a ser atingido, ou seja, diminuir a idade da conclusão do diagnóstico e da protetização.

Pode-se afirmar que foi possível atingir as metas de qualidade do Programa, devido à dedicação dos profissionais do próprio serviço, desde o processo de trabalho coletivo de implantação do programa, o envolvimento e comprometimento de todos os profissionais da rede de saúde do município, da regulação do serviço de saúde auditiva e da gestão com os outros equipamentos de saúde da cidade e de outros municípios, para o encaminhamento do neonato à realização da triagem e da busca ativa constante.
